# Design and analysis of a complementary structure-based high selectivity tri-band frequency selective surface

**DOI:** 10.1038/s41598-024-59712-1

**Published:** 2024-04-24

**Authors:** Zhiming Li, Xiaolong Weng, Xu Yi, Kai Li, Wei Duan, Mei Bi

**Affiliations:** 1 School of Electronic Science and Engineering, University of Electronic Science and Technology of China, Chengdu, 611731 China; 2 School of Materials and Energy, University of Electronic Science and Technology of China, Chengdu, 611731 China

**Keywords:** Metamaterials, Composites

## Abstract

This work presents a novel tri-band bandpass frequency selective surface (FSS) that achieves high-order filtering responses in different frequency bands by means of a complementary structure. The proposed FSS is composed of three metal periodic arrays, which are separated by multilayer dielectric substrates. The gridded-double convoluted loop (G-DCL) structure, which is the middle layer structure, is a hybrid resonator that generates different resonant frequencies. The top and bottom layer structures are designed as complementary structures to the middle layer. To accurately describe the frequency responses, an equivalent circuit model (ECM) has been constructed over the entire band from 0 to 16 GHz. The results of the simulation indicate that the developed FSS can generate three pass-bands operating at 3.79 GHz, 8.34 GHz, and 12.52 GHz, respectively, and − 3 dB fractional bandwidths are 52.8%, 13.7%, and 19.7%. The transmission responses at the edges of each passband show a quick roll-off from the passband to the stopband, and there is significant out-of-band suppression between adjacent passbands. Moreover, the FSS maintains excellent angular and polarization stability within a 50° range. For verification, the tri-band FSS has been fabricated and tested. The experimental results match the simulation results, validating the accuracy of the FSS design.

## Introduction

The FSS is composed of periodically arranged metallic patches or aperture elements, which exhibit distinct bandpass or band-stop filtering characteristics when interacting with electromagnetic waves^1,2^. Due to their excellent spatial electromagnetic filtering properties, FSS has been widely used in the development of microwave absorbers for electromagnetic stealth^3–7^ and FSS low-scattering radomes^8–10^. The goal of these applications is to reduce the radar cross-section (RCS) of sensitive targets, both military and civilian^11,12^. Traditionally, FSS are generally designed as 2-D periodic arrays of slot elements with a first-order filtering response^13,14^. However, as the satellite communications technology develops and the need for multi-frequency communication in military applications grows, the antenna's operating frequency gradually covers microwave and millimeter wave bands. To increase the capability of multifrequency antennas, there is a requirement for multiband FSS to have high-order filtering responses and sharp out-of-band suppression effects to satisfy antenna bandwidth and stealth requirements^15,16^.

Considerable work has been done to obtain multi-band high-order bandpass filtering responses. By cascading multiple gridded-loop structures, References^17–19^ designed a multi-band FSS with second-order filtering responses in each operating frequency band. However, the larger profile thickness and volume were the outcomes of the cascaded structure. Reference^20^ presented a novel method for designing second-order bandpass dual-band FSS that was based on sandwiching resonant elements between non-resonant elements in various metal layers. This method's primary characteristic is that the filter order strictly depends on the number of metal layers. A low-profile dual-band FSS with a second-order filtering response based on aperture-coupled patch resonators (ACPRs) was proposed in Reference^21^. Using three-dimensional (3D) cavity structures, dual-band bandpass FSS with high selectivity was realized in references^22,23^. The transmission response rapidly rolls off at the boundaries of the two passbands due to the three-dimensional (3D) cavity structure's ability to produce multiple transmission poles and zeros, and there is a significant electromagnetic wave suppression effect between the passbands. Additionally, by etching complementary frequency selective surface (CFSS) structures on both sides of the dielectric substrate and utilizing the coupling of the complementary layers, references^24–26^ were able to achieve multi-band designs. However, these CFSS only have first-order filtering responses in each operating frequency band with narrow bandwidths.

This paper proposes a tri-band FSS with high-order filtering responses based on complementary structures. It consists of G-DCL elements in the middle layer and complementary G-DCL elements in the top and bottom layers. Because of the complementarity of FSS elements, the three passbands are simple to design and implement and can be tuned individually. An equivalent circuit corresponding to the complementary structure is established to analyze its frequency behavior. Simulation and measurement results show that the proposed FSS can generate three pass-bands operating at 2.79–4.79 GHz, 7.77–8.91 GHz, and 11.29–13.75 GHz, respectively, and − 3 dB fractional bandwidths are 52.8%, 13.7%, and 19.7%. Each independent transmission band exhibits good polarization and angular stability. Moreover, steep roll-off and good reflection performance are achieved outside the passbands.

## Methods

### Tri-band FSS structural design

The metallic loop structure can be equivalent to a series LC resonator with impedance close to zero at its resonant frequency, which can generate transmission zeros. Conversely, the complementary structure of the metallic ring is a parallel LC resonator, which can produce a transmission pole^24^. Consequently, the resonance properties of the ring structure and its complementary structure are taken into consideration in this research. Ultimately, we use the G-DCL structure to design a three-frequency FSS with a high-order bandpass filtering response.

**Figure 1:**
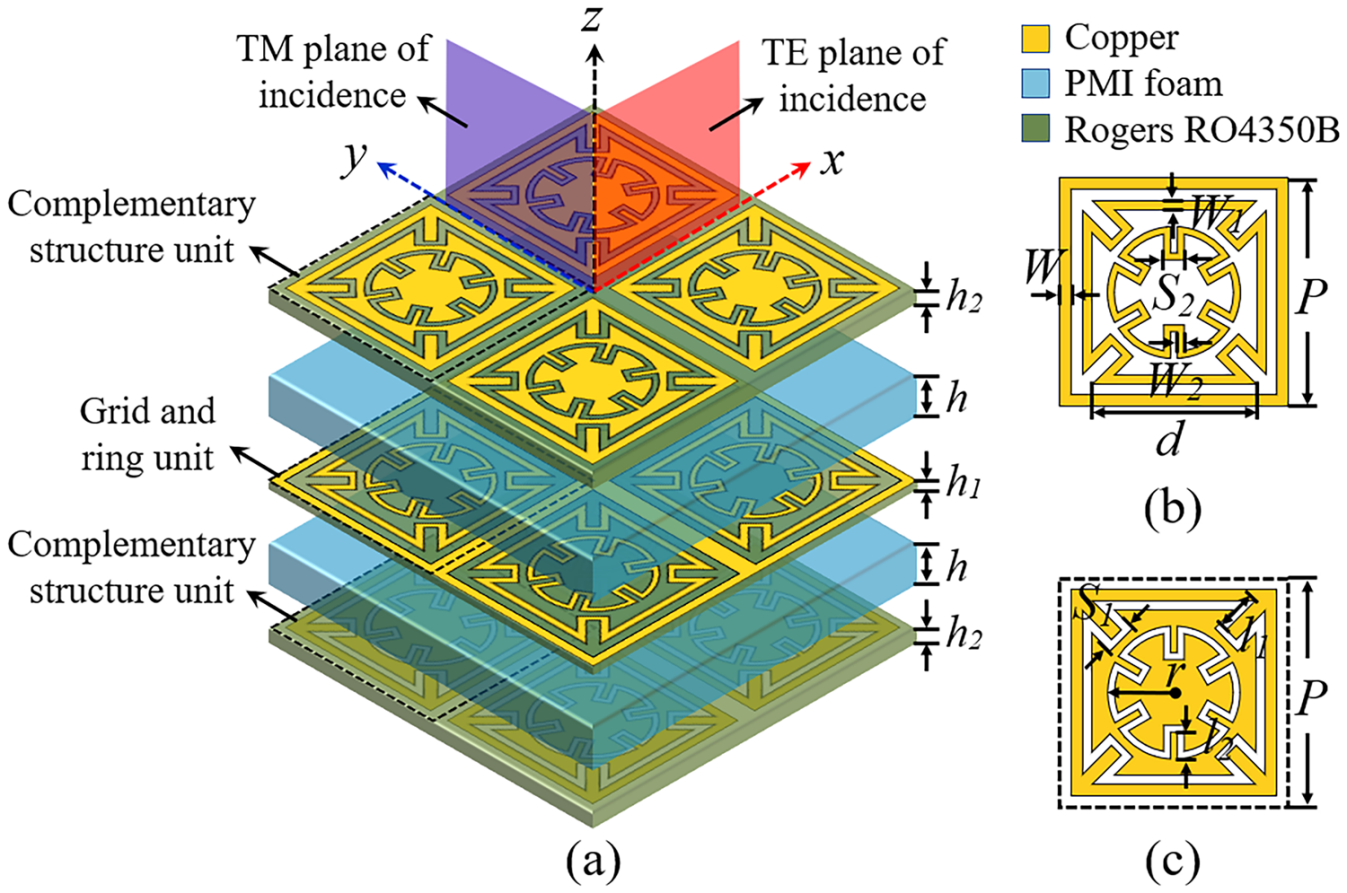
Design of the tri-band FSS element topology, (**a**) Three-dimensional perspective view, (**b**) G-DCL elements, (**c**) Complementary elements of G-DCL.

Figure 1a depicts the tri-band FSS's suggested structure. It consists of three FSS layers and multiple dielectric substrates. Two complex ring structures (a square ring and a circular ring) and a grid structure make up the middle layer of the FSS structure, known as the G-DCL structure. The G-DCL structure is depicted in Fig. 1b. The top and bottom layers of the FSS are complementary to the intermediate layer, as shown in Fig. 1c. All metal pattern materials are set to copper, with a conductivity of *σ* = 5.7 × 10^7^ S/m and a thickness of *t* = 0.035mm. The intermediate layer’s patterns are printed on a Rogers RO4350B substrate with a thickness of *h*_*1*_ = 0.168 mm, a relative permittivity of *ε*_*r*_ = 3.48, and a loss tangent of *tanδ* = 0.0037. The patterns of the top and bottom layers are printed on a Rogers RO4350B substrate with a thickness of *h*_*2*_ = 0.508mm. The PMI foam layer has a thickness of *h* = 3 mm, a loss tangent of *tanδ*_*1*_ = 0.001, and a relative permittivity of *ε*_*i*_ = 1.08. Table 1 contains the structural parameters of the proposed FSS.

**Table 1:** Relevant structural parameters of the tri-band FSS.

Parameter	Dimensions (mm)	Parameter	Dimensions (mm)
*P*	7.6	*r*	2.4
*D*	5.27	*S*_*1*_	1.2
*W*	0.25	*S*_*2*_	0.6
*W*_*1*_	0.2	*l*_*1*_	1.28
*W*_*2*_	0.2	*l*_*2*_	1

### Equivalent circuit model (ECM)

According to reference^25^, since the FSS on both sides of the dielectric plate are complementary in structure, the FSS element's ECM ought to be dual. Consequently, in this study, we first investigate the equivalent circuits of the middle layer G-DCL structure and ascertain the equivalent circuits of the top and bottom structures based on the dual relationship. Then, the ECM of the substrate and air layer is established using the transmission line theory. Ultimately, the ECM of the tri-band FSS is obtained by combining these ECMs.

**Figure 2:**
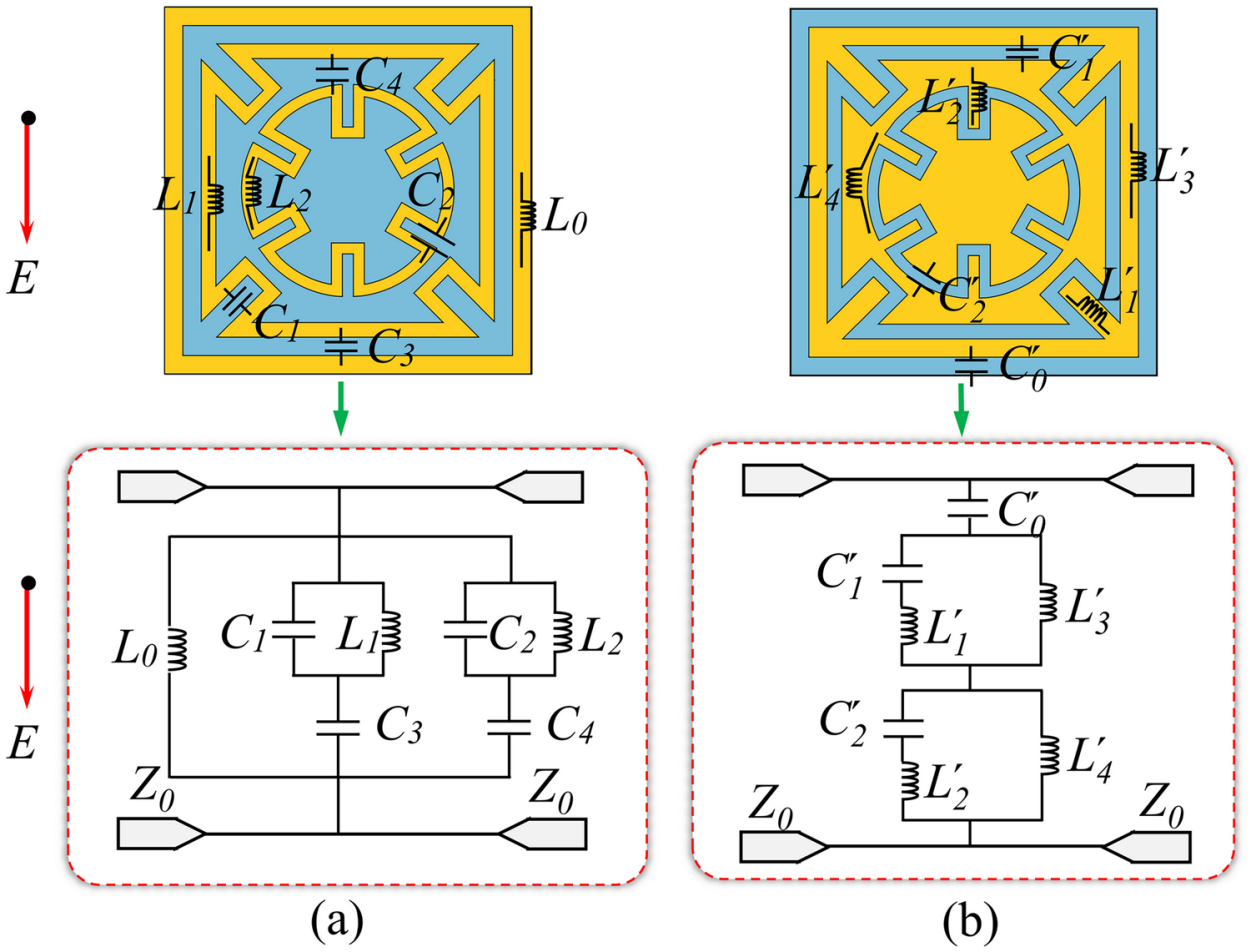
Equivalent circuits of the complementary elements, (**a**) Equivalent circuit of the G-DCL structure, (**b**) Equivalent circuit of the Complementary structure of G-DCL.

For the intermediate layer G-DCL elements, the grid structure can be equivalently represented as an inductance *L*_*0*_. Two hybrid resonators can be used for equivalent meandering square ring and circular ring structures, respectively. The square ring can be described as a series *L*_*1*_*C*_*3*_ resonator, with an additional parallel capacitor *C*_*3*_ introduced through the meandering part of the ring. Similarly, a hybrid resonator with capacitor *C*_*4*_ in parallel and *L*_*2*_ and *C*_*4*_ in series can be used to symbolize the ring structure. Through the above analysis, the ECM of the intermediate layer G-DCL structure is displayed in Fig. 2a.

Similarly, since the top and bottom FSS structures are complementary structures of the G-DCL structure, their ECM can be obtained quickly by using the dual form of the ECM of G-DCL. The gap capacitance between the two elements is represented by $C_{0}^{\prime }$. The external square-ring patch can be described as a parallel $C_{1}^{\prime } L_{3}^{\prime }$ resonator, and an additional parallel inductance $L_{1}^{\prime }$ is introduced through the extended branch part. The middle square patch can be equivalent as a parallel $C_{2}^{\prime } L_{4}^{\prime }$ resonator and an additional parallel inductance $L_{2}^{\prime }$*.* Therefore, an accurate equivalent circuit of the complementary structure of G-DCL is given in Fig. 2b.

**Figure 3:**
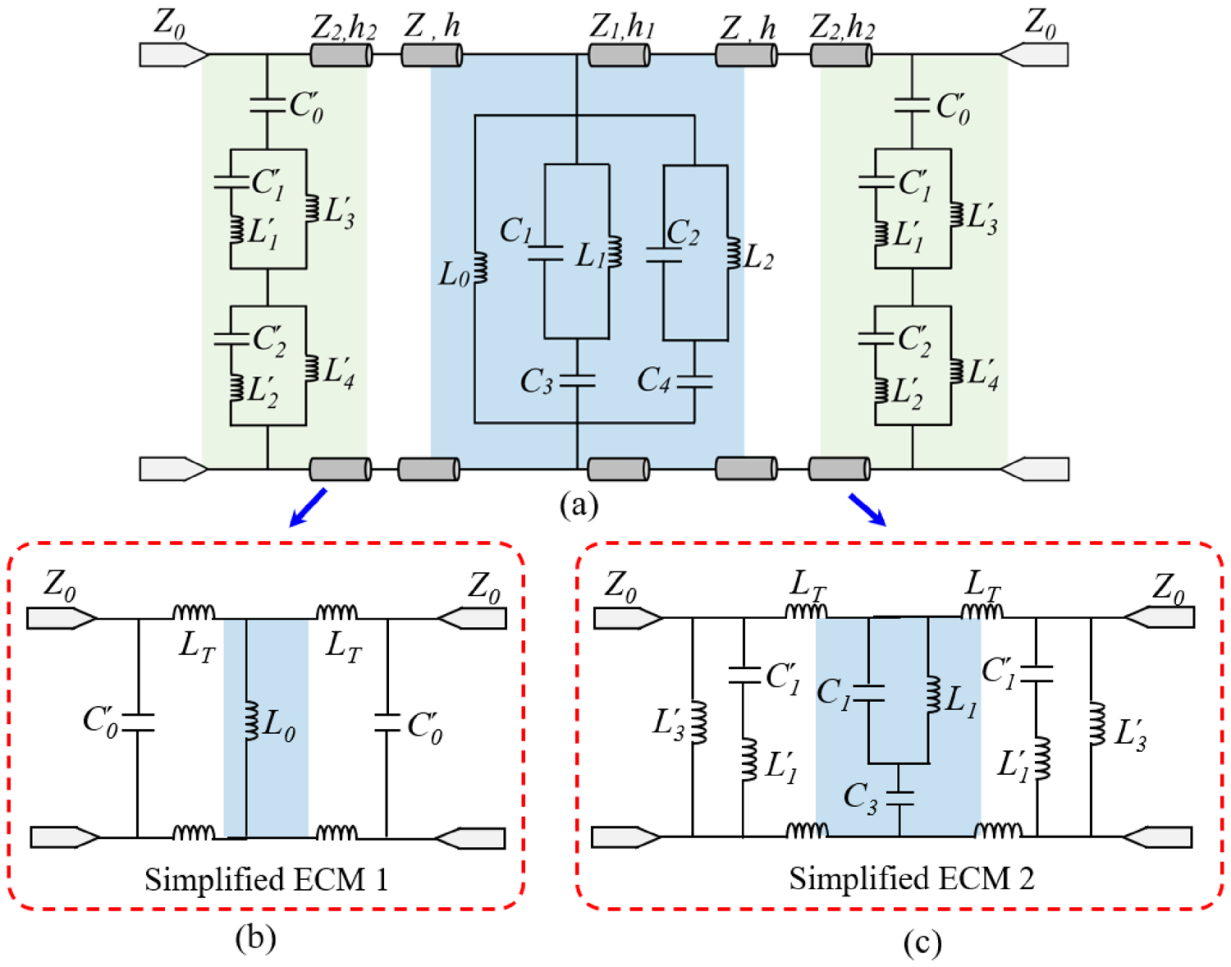
Equivalent circuits. (**a**) ECM of the tri-band FSS. (**b**) Simplified ECM in the first operating band. (**c**) Simplified ECM in the second or third operating band.

The remaining components of the FSS are modeled using transmission line theory. The air layer and each substrate can be modeled as transmission lines with characteristic impedances of *Z*_*0*_ = 377 Ω and *Z*_*i*_ = 377/$\sqrt {\varepsilon_{r} }$ (*i* = 1, 2, 3) Ω, respectively. Therefore, the final equivalent circuit structure of the proposed FSS is shown in Fig. 3a.

By analyzing the equivalent circuit shown in Fig. 3a, we can rapidly obtain the frequency response behavior of the proposed FSS. The central frequency of the first passband primarily depends on the length of the transmission line, the inductance *L*_*0*_, and the capacitance $C_{0}^{\prime }$. Figure 3b illustrates the simplified circuit model of the filter at the first passband, where the short transmission line (*h* <  < *λ*) is replaced by inductance *L*_*T*_. The equivalent inductance *L*_*T*_ of the short transmission line are denoted by the formula *L*_*T*_ = *μ*_*0*_*μ*_*r*_*h*. This circuit represents a second-order coupled bandpass filter (BPF) with inductive coupling. The central frequency *f*_*1*_ of the first passband can be calculated by

1 $$f_{1} \approx \frac{1}{{2\pi \sqrt {\left( {C^{\prime}_{0} + C_{T} } \right)\left( {L_{0} + L_{T} } \right)} }}$$

The two hybrid resonators and their dual circuits formed two third-order bandpass filters, which primarily determine the frequencies of the second and third passbands. Consequently, it is possible to simplify the ECMs of the second and third passbands, as illustrated in Fig. 3c. Equations (2) and (3) can be used to determine the center frequencies, or *f*_*2*_ and *f*_*3*_, of the second and third passbands, respectively.

2 $$f_{2} \approx \frac{1}{{2\pi \sqrt {\left( {C_{3} + C^{\prime}_{1} + C_{1} } \right)/\left( {L^{\prime}_{3} + L_{1} + L^{\prime}_{1} + L_{T} } \right)\left[ {C^{\prime}_{1} \left( {C_{3} + C_{1} } \right) + C_{T} \left( {C_{3} + C^{\prime}_{1} + C_{1} } \right)} \right]} }}$$

3 $$f_{3} \approx \frac{1}{{2\pi \sqrt {\left( {C_{4} + C^{\prime}_{2} + C_{2} } \right)/\left( {L^{\prime}_{4} + L_{2} + L^{\prime}_{2} + L_{T} } \right)\left[ {C^{\prime}_{2} \left( {C_{4} + C_{2} } \right) + C_{T} \left( {C_{4} + C^{\prime}_{2} + C_{2} } \right)} \right]} }}$$

Each hybrid resonator and its dual circuit will generate transmission zeros. As a result, the filter will generate four transmission zeros at frequencies *f*_*z1*_, *f*_*z2*_, *f*_*z3*_, and *f*_*z4*_ (where *f*_*z1*_ < *f*_*z2*_ < *f*_*z3*_ < *f*_*z4*_). The associated frequencies can be obtained by Eqs. (4)–(7).

4 $$f_{Z1} \approx \frac{1}{{2\pi \sqrt {L_{1} \left( {C_{1} + C_{3} } \right)} }}$$

5 $$f_{Z2} \approx \frac{1}{{2\pi \sqrt {C^{\prime}_{1} L^{\prime}_{1} } }}$$

6 $$f_{Z3} \approx \frac{1}{{2\pi \sqrt {L_{2} \left( {C_{2} + C_{4} } \right)} }}$$

7 $$f_{Z4} \approx \frac{1}{{2\pi \sqrt {C^{\prime}_{2} L^{\prime}_{2} } }}$$

It should be mentioned that a few forms similar to those given in^26,27^ can be used to establish the closed-forms between the FSS dimension and circuit parameters.

8 $$L_{0} \approx X_{0} /\omega = F\left( {P,2W,\lambda } \right)/\omega$$

9 $$L_{1} \approx 2\left( {d + 2l_{1} } \right)X_{0} X_{1} /\left[ {\omega P\left( {X_{0} + X_{1} } \right)} \right]$$

10 $$C_{3} \approx 2\varepsilon_{r} B_{1} d/\omega P$$

11 $$L_{2} \approx \pi rX_{2} /\omega P$$

12 $$C_{4} \approx 2\pi r\varepsilon_{r} B_{1} B_{2} /\left[ {\omega P\left( {B_{1} + B_{2} } \right)} \right]$$

13 $$C_{2} \approx \varepsilon_{0} \varepsilon_{r2} \frac{{6l_{2} }}{\pi }\log \left( {\frac{1}{{\sin \frac{{\pi W_{2} }}{{2l_{2} }}}}} \right)$$

14 $$C_{1} \approx \varepsilon_{0} \varepsilon_{r2} \frac{{4l_{1} }}{\pi }\log \left( {\frac{1}{{\sin \frac{{\pi W_{1} }}{{2l_{1} }}}}} \right)$$

Here, *X*_0_ = *F*(*P*, 2*W*, *λ*), *X*_1_ = *F*(*P*, 2*W*_1_, *λ*), *X*_2_ = *F*(*P*, 2*W*_2_, *λ*), *B*_1_ = 4*F*(*P*, *g*_1_, *λ*), and *B*_2_ = 4*F*(*P*, *g*_2_, *λ*). *λ* is the wavelength in the air at the operating frequency, *g*_1_ is the gap between the circular ring and the metal grid, and *g*_2_ is the gap between the circular ring and the square ring. Additionally, the factor *F* represents the normalized inductance or capacitance of the strip grating^26^.

15 $$F\left( {P,s,\lambda } \right) = \frac{P}{\lambda }\left[ {{\text{In}}\left( {\csc \frac{\pi s}{{2P}}} \right) + G\left( {P,s,\lambda } \right)} \right]$$

In the equation, *G* represents the correction term. Equations (8)–(15) illustrate the connection between the physical parameters of the proposed FSS structure and the ECM parameters. Nevertheless, these formulas might not produce accurate circuit element values because of the coupling effect of the multilayer structure. According to these approximate mapping relationships, we can quickly estimate the mapped FSS structural parameters and guide the structural design based on the circuit parameters. Table 2 displays the specific values of the circuit components.

**Table 2:** Relevant circuit parameters of ECM.

Parameter	Value	Parameter	Value
*C*_*1*_	0.01 pF	$L_{1}^{\prime }$	0.07 nH
*C*_*2*_	0.16 pF	$L_{2}^{\prime }$	0.0002 nH
*C*_*3*_	0.031 pF	$L_{3}^{\prime }$	0.63 nH
*C*_*4*_	0.071 pF	$L_{4}^{\prime }$	0.53 nH
$C_{0}^{\prime }$	0.22 pF	*L*_*0*_	2.55 nH
$C_{1}^{\prime }$	0.56 pF	*L*_*1*_	2.8 nH
$C_{2}^{\prime }$	0.33 pF	*L*_*2*_	1.3 nH
*Z*	362.8 Ω	*Z*_*1*_	202.1 Ω
*Z*_*2*_	202.1 Ω	*L*_*T*_	3.76 nH

### Simulation results and discussion

In order to simulate an infinite array, the unit cell of the tri-band FSS is configured with a periodic boundary condition. This choice of periodic boundary condition enables the simulation to accurately replicate the repetitive arrangement of unit structures within the tri-band FSS and effectively achieves a comprehensive analysis of the proposed structure's performance.

**Figure 4:**
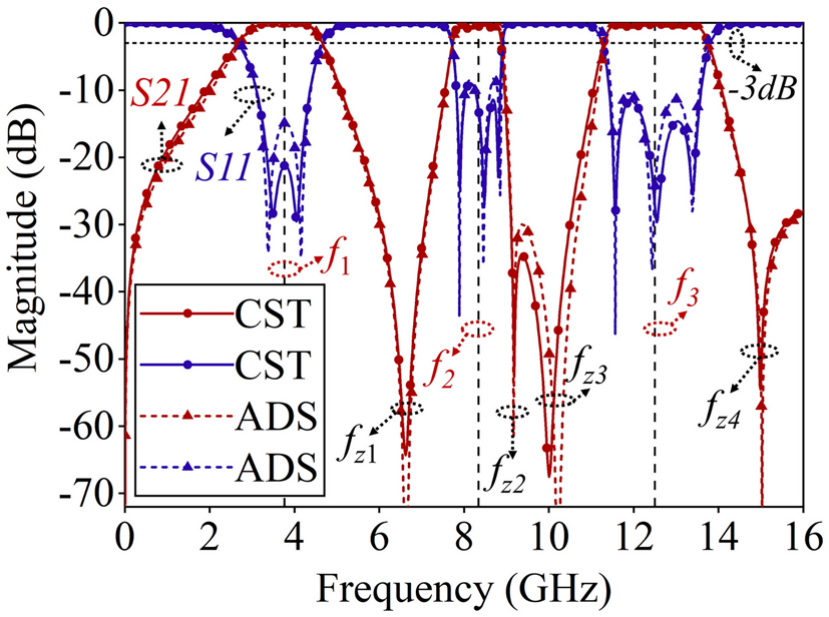
Frequency response curve of the tri-band FSS.

Figure 4 displays the S-parameters (electromagnetic wave incident vertically) derived from full-wave simulation and ECM calculation. It is evident that the proposed FSS structure exhibits three passbands that are highly selective. These passbands have − 3 dB bandwidths of 2 GHz (2.79–4.79 GHz), 1.14 GHz (7.77–8.91 GHz), and 2.46 GHz (11.29–13.75 GHz), respectively. At frequencies *f*_z1_ = 6.62 GHz, *f*_z2_ = 9.18 GHz, *f*_z3_ = 10 GHz, and *f*_z4_ = 15.1 GHz, there are four distinct transmission zeros. These transmission zeros result in a rapid transition from transmission to reflection at the edges of each passband. Additionally, the ECM computations' outcomes agree well with the full-wave simulations, thus validating the accuracy of the established ECM.

### Surface current distribution

**Figure 5:**
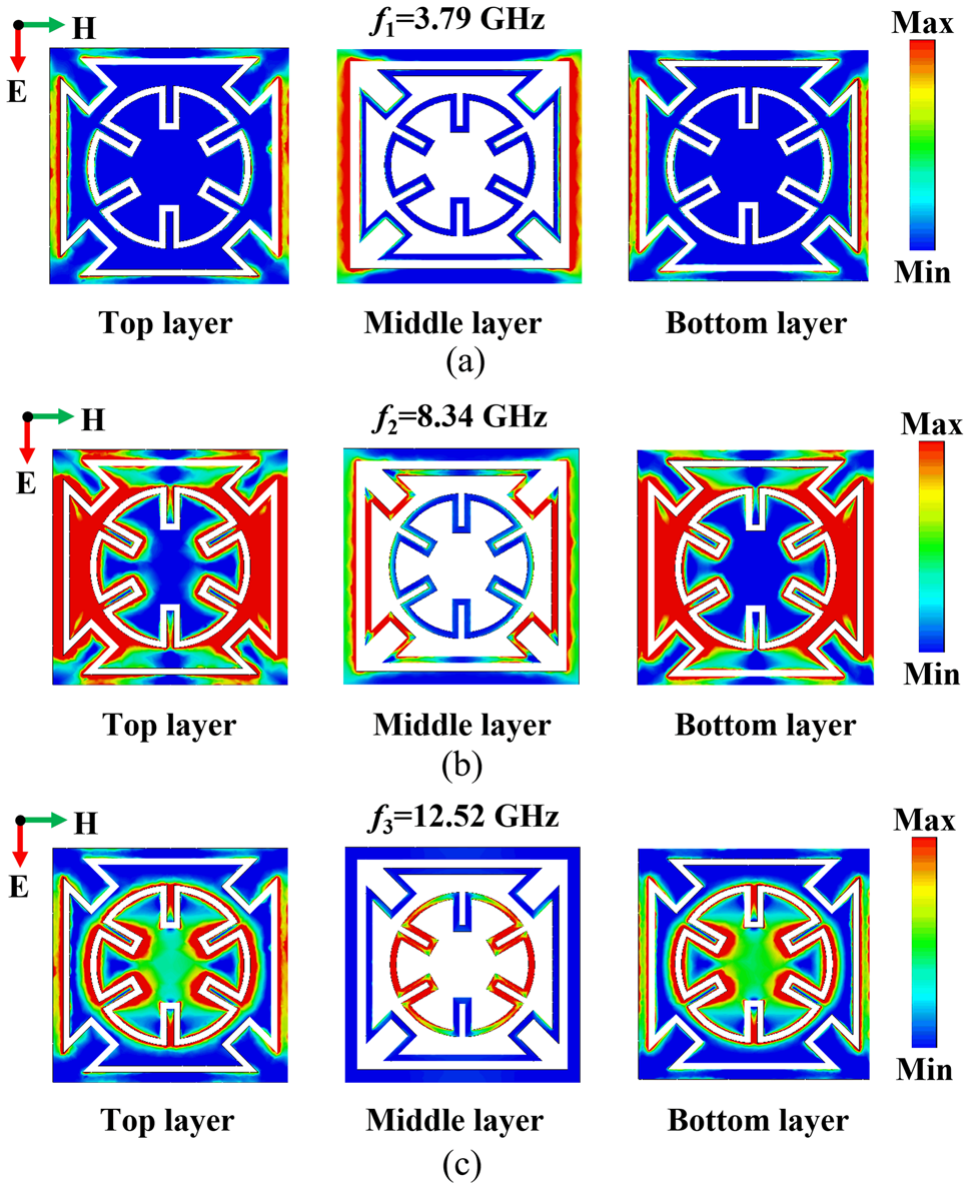
Surface current distribution at different frequency points. (**a**) Surface current distribution 1 (*f*_*1*_ = 3.79 GHz). (**b**) Surface current distribution 2 (*f*_*2*_ = 8.34 GHz). (**c**) Surface current distribution 3 (*f*_*3*_ = 12.52 GHz).

To gain a better understanding of the proposed FSS structure's working mechanism, Fig. 5 shows the surface current distribution at different passband frequencies. When electromagnetic waves are vertically incident on the FSS structure, it is observed that the surface currents mainly concentrate on the intermediate layer line grid structure and its corresponding complementary structure at *f*_1_ = 3.79 GHz. This means that the coupling resonance between the intermediate layer grid structure and its complementary structures above and below forms the first passband. At *f*_2_ = 8.34 GHz, the surface currents primarily gather on the meandering square ring structure, while at *f*_3_ = 12.52 GHz, they mainly focus on the meandering circular ring structure. This indicates that the coupling resonance between the square ring and circular ring structures and their complementary structures primarily determines the second and third passbands. The law of surface current distribution is consistent with the conclusion of equivalent circuit; it can be observed that the established ECM accurately describes the working mechanism of the tri-band FSS structure.

**Figure 6:**
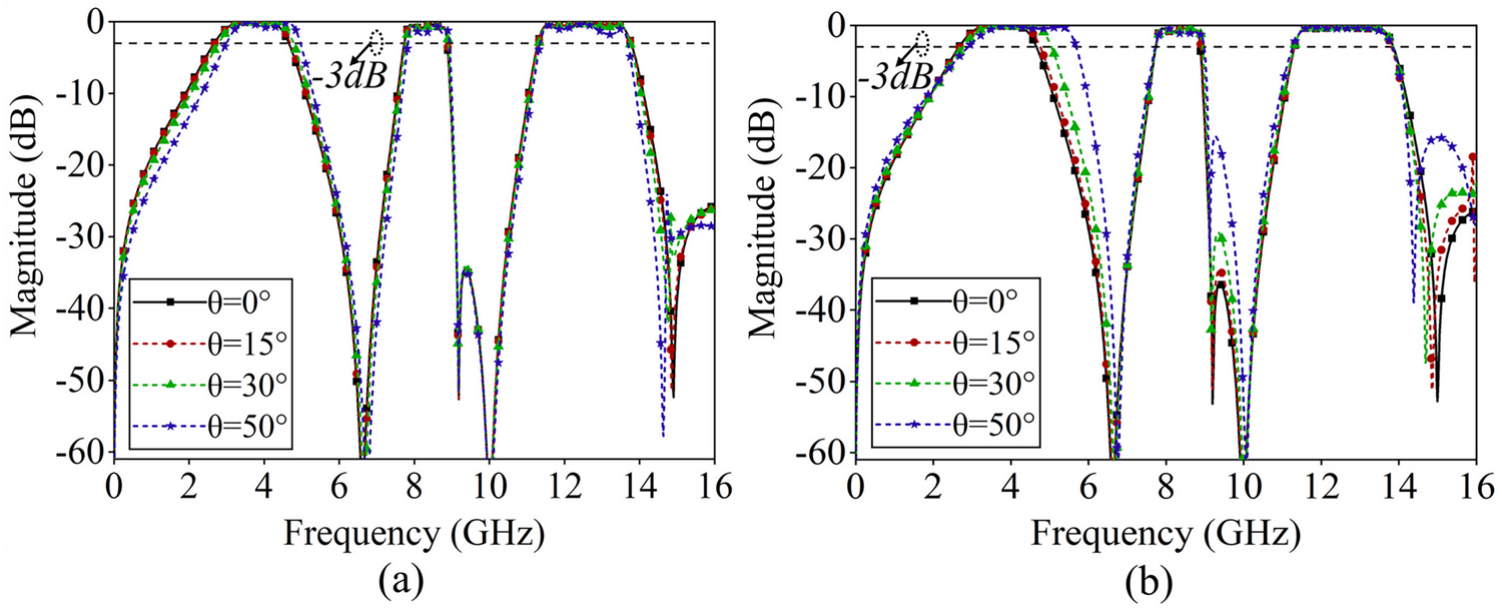
Transmission performance of the tri-band FSS at oblique incidence, (**a**) TE polarization, (**b**) TM polarization.

Figure 6 shows the tri-band FSS's transmission characteristics under the oblique incidence of TE and TM polarized waves. As the incident angle increases, the designed FSS structure exhibits stable transmission performance, maintaining high transmittance across all three passbands. Notably, for TE polarization, the insertion loss within the passbands increases slightly as the electromagnetic wave incident angle increases from 0° to 50°. Furthermore, for TM polarization, there is a discernible shift in the first passband towards higher frequencies as the incident angle increases.

**Figure 7:**
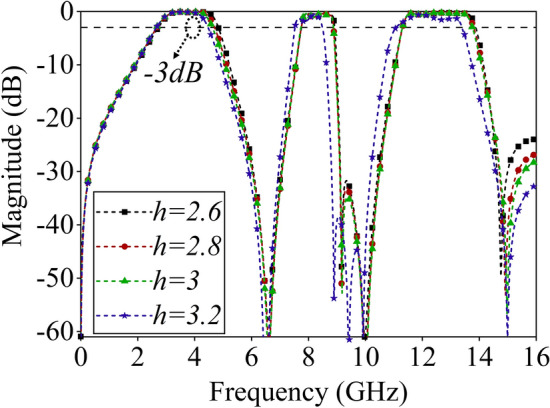
Transmission coefficients of the tri-band FSS for different thickness of PMI form layers.

The transmission coefficient of the tri-band FSS under PMI foam layers with different thicknesses is shown in Fig. 7. With the increase in *h*, the frequency response of the three passbands remains relatively stable, the bandwidth of each passband is slightly reduced. When the thickness of the foam layer increases, the equivalent inductance *L*_*T*_ of the transmission line increases, which leads to the resonance frequency moving to a low frequency. Therefore, the transmission poles/zeros are closer, which reduces the bandwidth.

To clarify the advantages of the proposed FSS, we conducted a comparative study with recently published designs of multi-band FSS. As indicated in Table 3, our proposed structure demonstrates the advantages of a high-order filtering response. Additionally, the comparison shows better results in terms of compactness, reduced profile, high angular stability, and wide passbands.

**Table 3:** Comparison with recently published results.

Reference	Number of in-band poles	− 3 dB FBW	Stability	Overall thickness (*λ*_*0*_)	Element size (*λ*_*0*_)
^14^	2/2	5.8% / 3.5%	Up to 40°	0.04*λ*_*0*_	0.8*λ*_*0*_
^22^	2/2	10.4% / 6.2%	Up to 40°	0.13*λ*_*0*_	0.17*λ*_*0*_
^16^	1/2/2	3.1% / 5.7% / 5.1%	Up to 60°	0.07*λ*_*0*_	0.16*λ*_*0*_
^17^	1/2/2	13.6% / 22.7% / 12.6%	Up to 60°	0.03*λ*_*0*_	0.17*λ*_0_*0.15*λ*_*0*_
^18^	2/2/2	31.1% / 12.8% / 7.1%	Up to 45°	0.14*λ*_*0*_	0.27*λ*_*0*_
^19^	1/1/1	12.8% / 15.3% / 5.6%	Up to 45°	0.06*λ*_*0*_	0.14*λ*_*0*_
**This Work**	**2/3/3**	**52.8% / 13.7%/ 19.7%**	**Up to 50°**	**0.092*λ***_***0***_	**0.096*λ***_***0***_

Significant values are in bold.

### Experimental validation

**Figure 8:**
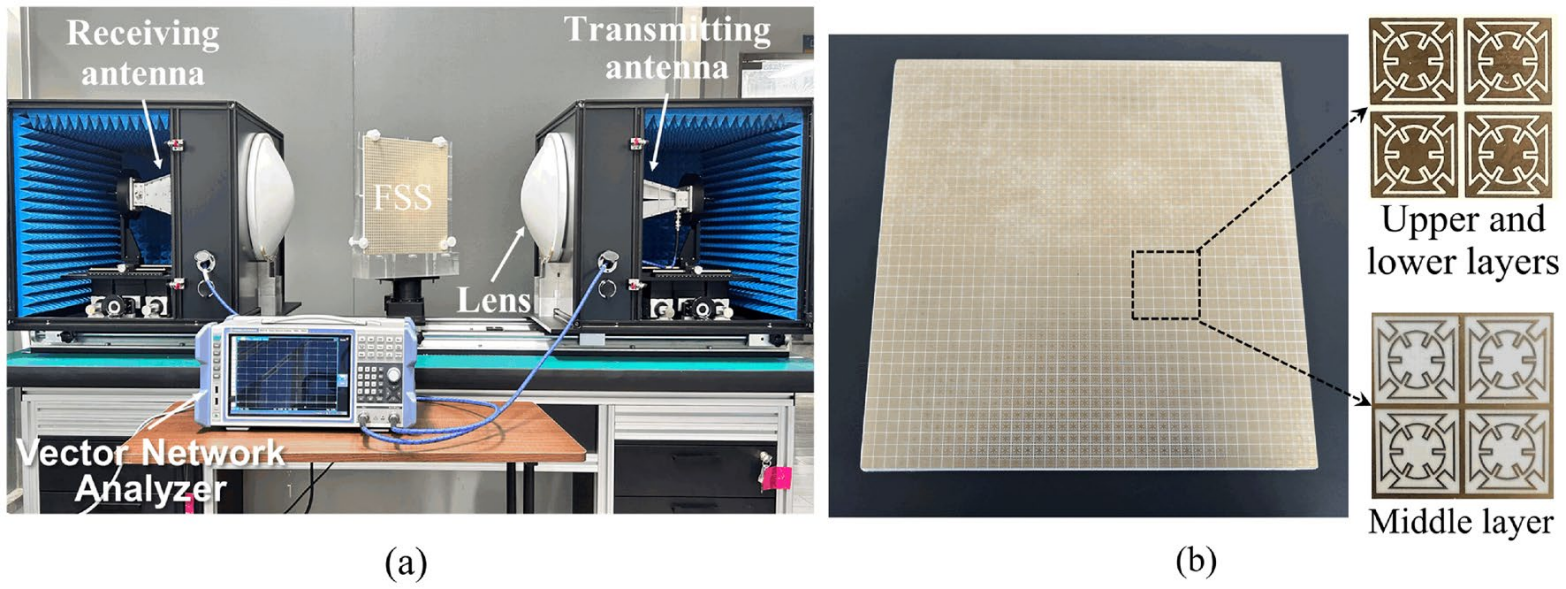
Transmission characteristic measurement, (**a**) Experimental setup, (**b**) Prototype of the tri-band FSS.

To validate the wideband transmission characteristics of our proposed design, a prototype of the tri-band FSS was fabricated using printed circuit board technology (PCB) according to the design parameters for testing. As shown in Fig. 8a, the fabricated FSS sample has dimensions of 304 mm × 304 mm and consists of 40 × 40 FSS units. The patterns of each layer were printed on a substrate made of Rogers RO4350B (*ε*_*r*_ = 3.48 and *tanδ* = 0.0037). Two PMI foam (*ε*_*r*_ = 1.08 and *tanδ*_*1*_ = 0.001) with a thickness 2.0 mm has been used as the low-dielectric substrate. The geometric parameters of the FSS were set to the values specified in Table 1.

The experimental setup for transmission characteristics is illustrated in Fig. 8b. The FSS was fixed on a rotatable support, and measurements were conducted using a pair of focusing lenses, a pair of horn antennas, and an Agilent Technologies N5235B vector network analyzer. To ensure accurate testing and minimize the influence of the testing system and environment, the transmission characteristics without the FSS were initially measured for calibration. Subsequently, the transmission characteristics of the FSS prototype were measured. Finally, the obtained transmission coefficients were normalized to obtain the final test results.

### Transmission performance

**Figure 9:**
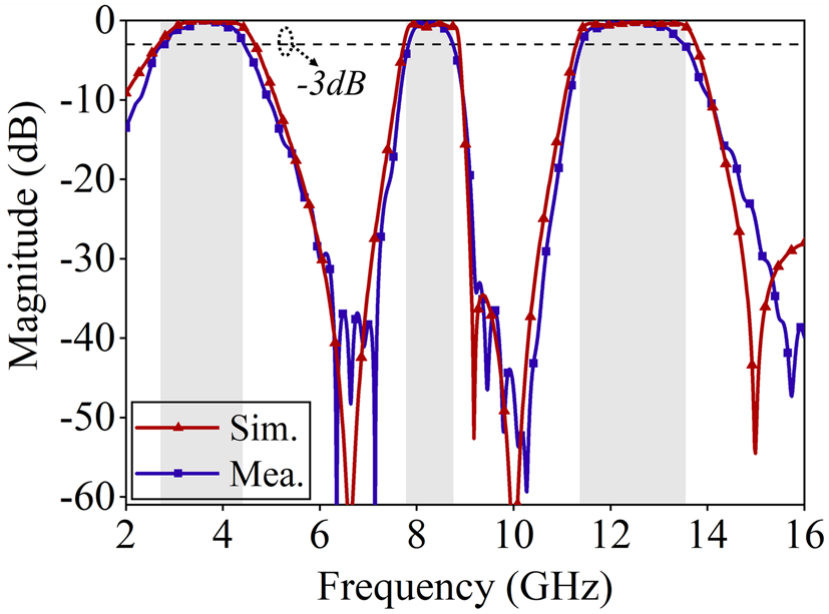
Comparison of FSS simulation and test results.

**Figure 10:**
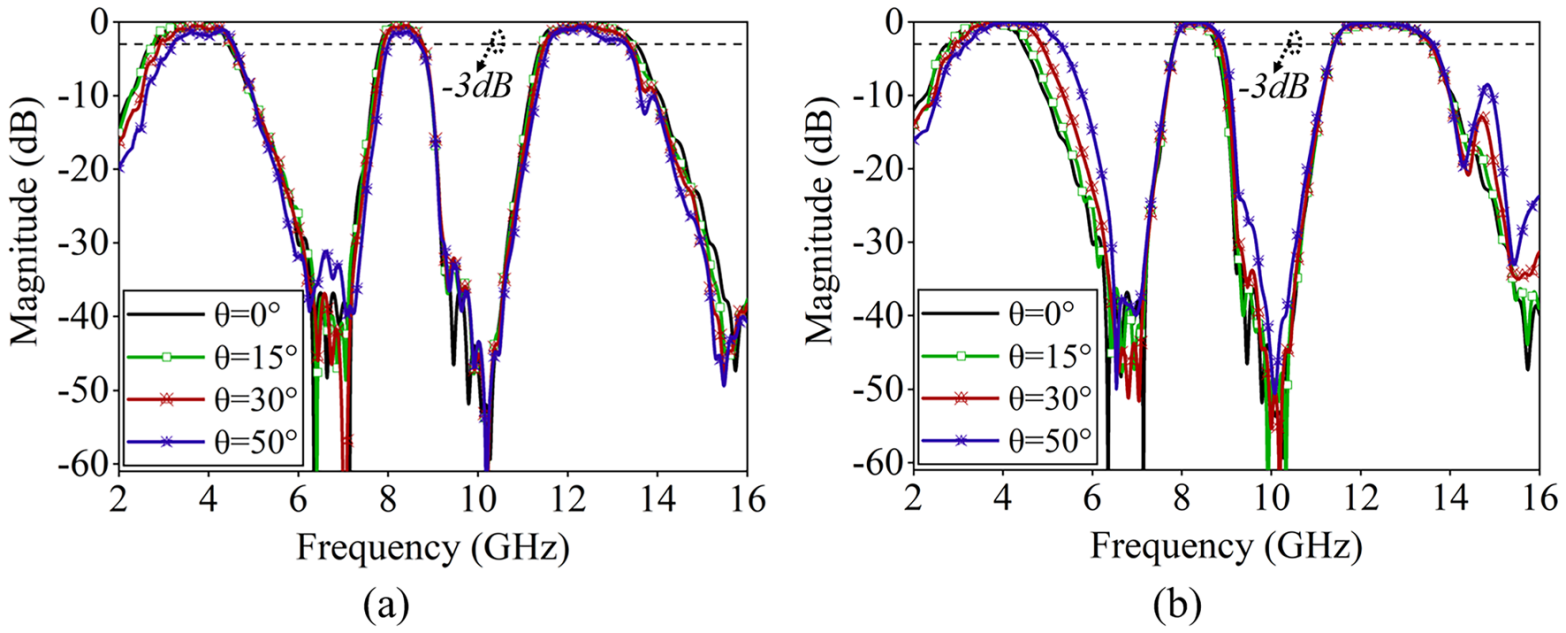
Test results of S21 curves at different incidence angles, (**a**) TE polarization, (**b**) TM polarization.

Figure 9 illustrates the simulated and measured transmission coefficients of the proposed FSS under vertical incidence. The FSS exhibits three passbands of 2.79–4.55 GHz, 7.83–8.81 GHz, and 11.32–13.48 GHz, with simulation results in good agreement with the experimental results. The FSS demonstrates wideband tri-band filtering characteristics and rapid edge roll-off characteristics. Additionally, the transmission coefficients of the tri-band FSS were tested at different incident angles, as shown in Fig. 10. Within the range of 0–50° of incident angle, the insertion loss of the three passbands remains within − 3 dB, confirming the excellent angular stability of the proposed FSS.

## Conclusion

This work presents a tri-band FSS with high-order bandpass responses achieved through the cascading of complimentary hybrid resonant elements. The − 3dB transmission bandwidths are 2.79–4.79 GHz, 7.77–8.91 GHz, and 11.29–13.75 GHz, respectively, with each passband edge exhibiting rapid roll-off characteristics. Furthermore, the working mechanism of the FSS structure is analyzed using an ECM, and prototype samples of the FSS are fabricated and tested. The experimental results are in good agreement with the simulation results, demonstrating satisfactory stability under different polarization and incident angles.

## Data Availability

The raw/processed data are being used by ongoing research projects, thus these findings cannot be shared at this time. If necessary, please contact the corresponding author: bimei@uestc.edu.cn.
